# The emerging roles of LINC00511 in breast cancer development and therapy

**DOI:** 10.3389/fonc.2024.1429262

**Published:** 2024-08-14

**Authors:** Lifeng Zhao, Sangita Biswas, Yepeng Li, Suren Rao Sooranna

**Affiliations:** ^1^ Department of Oncology, Affiliated Hospital of Youjiang Medical University for Nationalities, Baise, China; ^2^ Faculty of Medicine, MAHSA University, Jenjarom, Selangor, Malaysia; ^3^ Department of Preclinical Sciences, Faculty of Dentistry, MAHSA University, Jenjarom, Selangor, Malaysia; ^4^ Department of Metabolism, Digestion and Reproduction, Imperial College London, Chelsea and Westminster Hospital, London, United Kingdom; ^5^ Life Science and Clinical Research Center, Youjiang Medical University for Nationalities, Baise, China

**Keywords:** lncRNAs, LINC00511, breast cancer, mechanisms, biological functions

## Abstract

Breast cancer (BC) is associated with malignant tumors in women worldwide with persistently high incidence and mortality rates. The traditional therapies including surgery, chemotherapy, radiotherapy and targeted therapy have certain therapeutic effects on BC patients, but acquired drug resistance can lead to tumor recurrence and metastasis. This remains a clinical challenge that is difficult to solve during treatment. Therefore, continued research is needed to identify effective targets and treatment methods, to ultimately implement personalized treatment strategies. Several studies have implicated that the long non-coding RNA LINC00511 is closely linked to the occurrence, development and drug resistance of BC. Here we will review the structure and the mechanisms of action of lnc RNA LINC00511 in various cancers, and then explore its expression and its related regulatory mechanisms during BC. In addition, we will discuss the biological functions and the potential clinical applications of LINC00511 in BC.

## Graphical Abstract

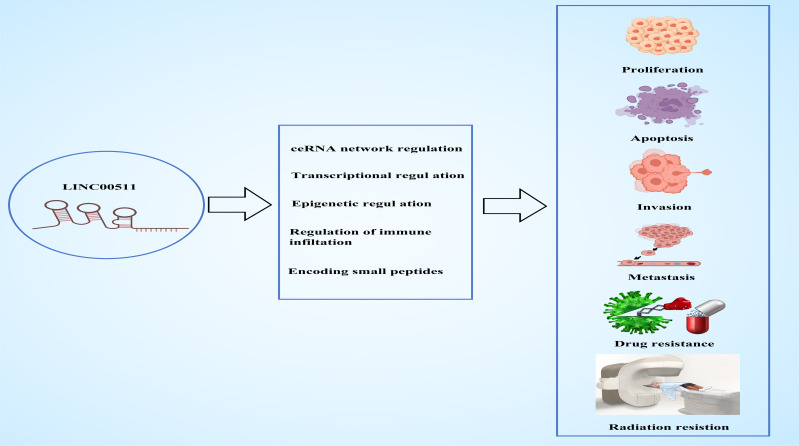

## 1 Introduction

Although governments and health organizations around the world have taken a series of measures and made great efforts with regards to cancer prevention and treatment, the incidence and mortality rates for breast cancer (BC) have not been effectively controlled. These continue to show an increasing trend (1). Cancer data statistics show that in 2020, there were about 2.3 million newly diagnosed cases of BC. As a result, BC has surpassed lung cancer (LC) for the first time, and has become the leading cancer among women (2). Currently, BC remains one of the main causes of death in women worldwide, posing a serious threat to the health and lives (3). There are currently several ways to classify BC. Based on genetic and epigenetic characteristics, breast cancer cell lines can be classified as luminal A, luminal B, HER2 positive, triple negative A and triple negative B (4) Clinically, BC can be classified into estrogen receptor (ER)-positive and ER-negative as well as HER2 (human epidermal growth factor receptor 2)-positive and HER2-negative subtypes. This classification is based on the levels of ER, progesterone receptor (PR) and HER2 present (5, 6).

Although medical professionals have conducted extensive and long-term research on the diagnosis and treatment of BC, as yet no significant breakthroughs have been achieved. Therefore, the therapeutic effects for BC patients have not improved significantly, and the survival times have not been significantly extended. To date, the conventional treatment methods for BC are primarily surgery and targeted therapies using drugs, radiography, hormones and antibodies (7). Although the emerging targeted therapies have brought hope to BC patients in recent years, cellular drug resistance leading to tumor recurrence and metastasis has posed a severe challenge to its treatment. Therefore, continued research to identify effective targets and treatment methods are in demand from BC patients as well as health professionals. In this context, the role of long non-coding RNAs (lncRNAs) in cancers has been the subject of intense research from scholars, worldwide.

LncRNAs are transcripts that contain more than 200 nucleotides. Due to their lack of long open reading frames, these molecules were previously assumed to have no protein-coding ability. However, with the continuous development of medical testing techniques, there is now a substantial body of evidence to show that some lncRNAs have these abilities (8–11). The discovery that lncRNAs can encode proteins has drawn widespread attention to their biological roles and have made them a research hotspot in recent years.

Studies have shown that lncRNAs can regulate the expression and function of various genes through multiple mechanisms. These include regulation of chromatin remodeling, splicing, mRNA transcription, DNA methylation, mRNA stability and translation and post-transcriptional regulation (12–17). In addition, mature lncRNAs can also bind to RNA/DNA binding proteins, transcription factors (TFs), chromatin modification complexes, RNA transcripts, mature mRNAs, microRNAs, DNA and chromatin) to form supramolecular structures (18). This can lead to regulation of the expression and function of target genes. Numerous studies implicate lncRNAs in the regulation of essential biological and cellular processes. These include proliferation, differentiation, invasion, migration, angiogenesis, stemness, epithelial-mesenchymal transition, cell apoptosis, immune responses and tumor treatment resistance in malignant tumors (19–21).

Therefore, dysregulation of either their expression or function can lead to pathological and physiological changes in the human body. These can result in the development of abnormalities and malignancy resulting in diseases. In recent years, the functional roles of lncRNAs in malignant tumors have gradually been revealed. Several studies have shown that lncRNAs are abnormally expressed in malignant tumors such as renal cell carcinoma, gastric cancer, liver cancer, non-small cell LC, colorectal cancer, glioblastoma, osteosarcoma and ovarian cancer (22–31). They have been shown to be implicated in the regulation and development of cancers.

Long intergenic non-coding RNAs (lincRNAs) can be considered to be lncRNAs as they share similarities of structure and function. Although lincRNAs do not participate in encoding specific proteins, they can act as regulatory factors to regulate the expression of target genes and thus play roles in various cellular and biological processes (32). Existing studies have shown that dysregulation of lincRNA expression and function can also lead to the occurrence and progression of tumors (33–36).

LINC00511 is a newly discovered lncRNA and it is known to be associated with LC (37, 38). It has also been reported to be abnormally expressed in many types of malignant tumors where it can accelerate tumor progression. It inhibits malignant cell apoptosis and promotes the proliferation, migration, invasion, metastasis and chemotherapy resistance of tumor cells (39–41). Therefore, LINC00511 is a potential cancer biomarker and a promising therapeutic target. Currently, there have been multiple reports on the link between LINC00511 and BC, but the existing research directions are scattered and there is a lack systematic summarization. Therefore, we reviewed the expression, structural characteristics, mechanisms and functional roles of LINC00511 in BC with a view to clarifying its clinical significance and therapeutic potential.

## 2 Structural characteristics of LINC00511

**Figure 1:**
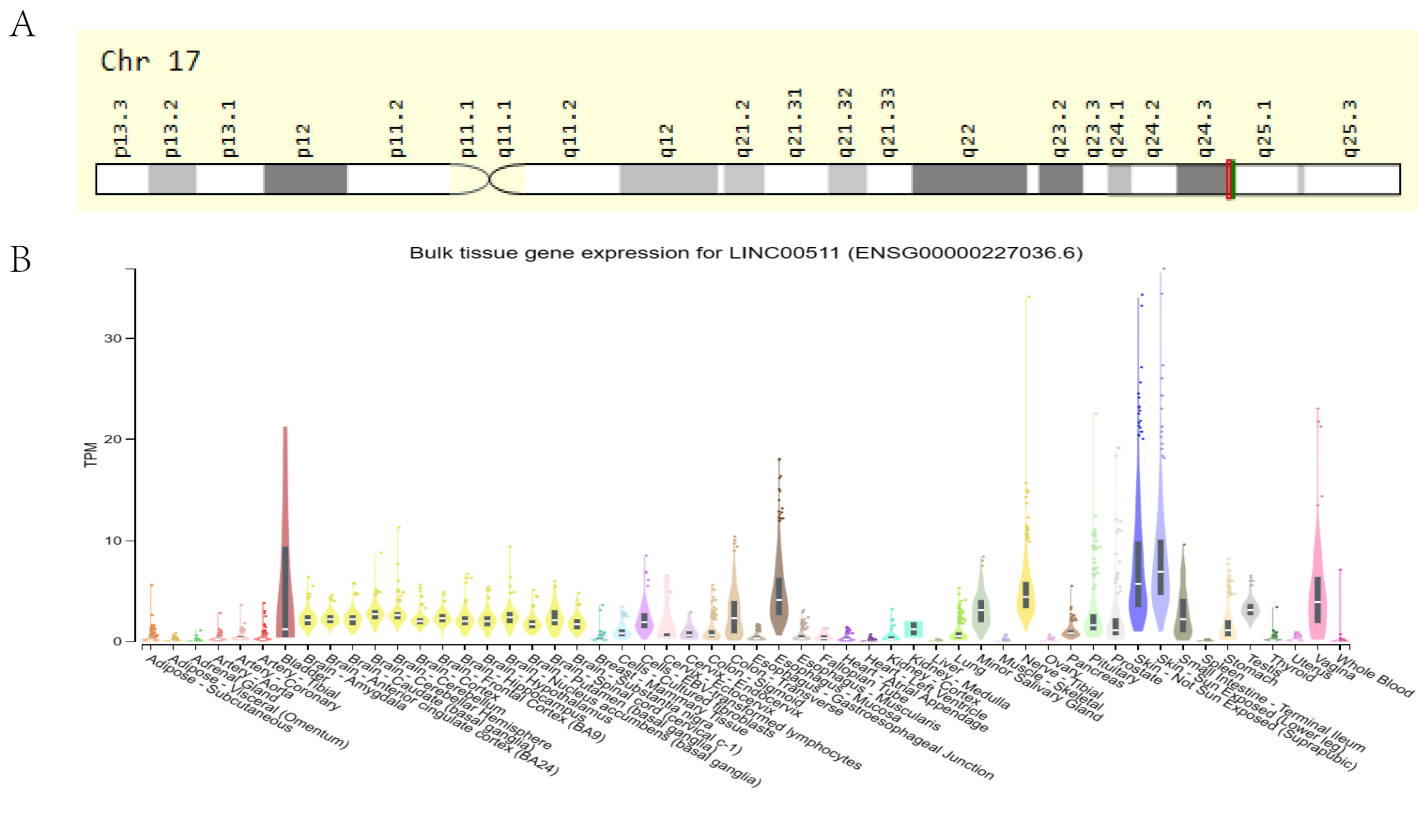
**(A)** Localization of LINC00511 in the human chromosome. **(B)** The expression of LINC00511 in normal human tissues from information obtained from the GTEx database.

Cabanski et al. first reported LINC00511, which is also known as onco-LncRNA-12, in 2015 after a pan-cancer transcriptome analysis (42). It is located on the negative strand of 17q24.3 region, 72,290,091-72,640,472 (GRCh38/hg38), and it has been allocated the transcript number, ENST00000453722.6 (**Figure 1A**). It is 1716nt in length and consists of 5 exons (43). Studies have identified 107 alternative splice variants of LINC00511. Except for the two splicing variants, LINC00511-279 and LINC00511-278, which retain the introns, the others belong to the lncRNA transcriptome (https://asia.ensembl.org/Homo_sapiens/Gene/Summary?db=core;g=ENSG00000227036;r=17:72290091-72640472). The NIH Genotype-Tissue Expression (GTEx) project (https://commonfund.nih.gov/GTEx, accessed on April 9, 2024) suggested that LINC00511 is widely expressed in normal human tissues (**Figure 1B**). However, its expression is highest in the skin, vagina and esophagus, suggesting that its presence in these organs has biological significance.

## 3 Functional roles of LINC00511

**Figure 2:**
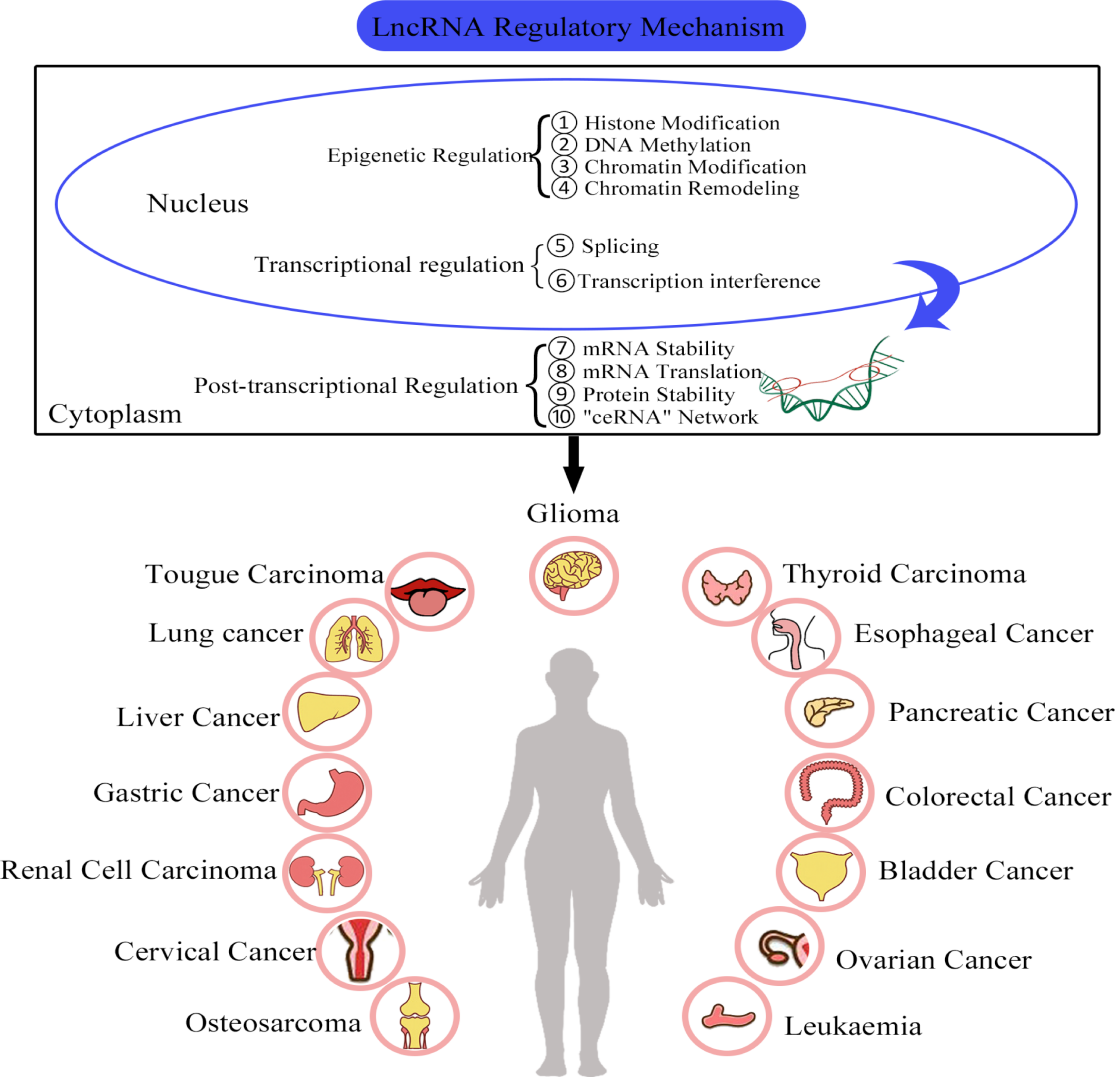
The functional roles and regulatory mechanisms of lncRNAs in various cancers. ① LncRNAs are involved in the regulation of histone modification. ② LncRNAs are involved in DNA methylation. ③ LncRNAs can regulate chromatin modification. ④ LncRNAs can bind to chromatin re-modelling factors and regulate gene expression. ⑤ LncRNAs can interact with splicing factors. ⑥ LncRNAs can cause transcription factors to interfere with gene transcription. ⑦ LncRNAs can regulate RNA stability. ⑧ LncRNAs can participate in the regulation of RNA translation. ⑨ LncRNAs are involved in the regulation protein stability. ⑩ LncRNAs can act as miRNA sponges.

Studies have shown that the biochemical functions and regulatory mechanisms of lncRNAs are closely related to their genomic locations and subcellular localizations (44). For example, lncRNAs that are located in the cell nuclei are mainly associated with regulation in epigenetic modifications and transcriptional processes. However, cytoplasmic lncRNAs typically exert their regulatory effects through post-transcriptional mechanisms such as the regulation of mRNA stability, protein translation and competition with endogenous RNA (ceRNA) networks (9, 45, 46) (**Figure 2**).

With respect to LINC00511, studies have shown that its intracellular localization varies in different cancers. Wu et al. used subcellular fractionation and FISH assays and they localized LINC00511 primarily in the cytoplasm in lung squamous cell carcinoma (46). However, in ovarian cancer it was localized in the nuclei of ovarian cancer cells (47). Zhao et al. found that LINC00511 was abundant in both the cytoplasm and nuclei of pancreatic cancer cells by using FISH (48). The widespread subcellular localization of LINC00511 differs in different types of tumor cells and this is likely to reflect on its biological functions and regulatory mechanisms (**Table 1**).

**Table 1:** The functions and regulatory mechanisms associated with LINC00511 in various malignant tumors.

Tumor type	Molecular function	Mechanism of action	Biological function	References
Glioma	ceRNA network regulation	Sponge effect of miR-126-5p and activation of Wnt/β-catenin	Promotion of glioma cell resistance to temozolomide	(49)
	ceRNA network regulation	Sponge effect of miR-15a-5p and activation of AEBP1	Regulation of glioma cell proliferation and migration	(50)
	ceRNA network regulation	Sponge effect of miR-524-5p and activation of YB1/ZEB1	Promotion of glioblastoma cell proliferation, migration and ETM	(51)
	ceRNA network regulation	Sponge effect of miR-124-3p and activation of CCND2	Accelerated proliferation and invasion of glioma cells	(52)
Non-small cell lung cancer (NSCLC) Non-small cell lung cancer (NSCLC)	Epigenetic regulation	Inhibition of LATS2 and KLF2 expression by binding EZH2 and LSD1	Promotion of NSCLC cell proliferation and invasion	(53)
	Epigenetic regulation	Inhibition of P57 expression by binding to EZH2	Promotion of NSCLC cell proliferation and invasion	(54)
	ceRNA network regulation	Sponge effect of miR-625-5p and activation of PKM2	Promotion of lung adenocarcinoma (LA) cell proliferation, invasion and migration	(55)
	ceRNA network regulation	Sponge effect of miR-195-5p and activation of GCNT3	Promotion of LA cell proliferation, invasion and migration	(56)
	ceRNA network regulation	Sponge effect of miR-625-5p and activation of GSPT1	Promotion of LA cell proliferation, invasion and migration	(57)
	ceRNA network regulation	Sponge effect of miR-150-5p and activation of TADA1	Promotion of lung squamous cell carcinoma progression	(46)
	ceRNA network regulation	LINC00511 silencing promotes miR-497-5p expression to inhibit SMAD3	Increased radio-sensitivity of LA cells	(58)
	ceRNA network regulation	Sponge effects of miR-126-5p and miR-218-5p, upregulation of COL1A1 and activation of PI3K/AKT	Promotion of LA cell proliferation and metastasis	(59)
	ceRNA network regulation	Sponge effect of miR-625-5p and activation of PKM2	Promotion of LA cell proliferation and metastasis	(55)
	ceRNA network regulation	Silence of LINC00511, promotion of miR-182-3p expression and inhibition of BIRC5	Inhibition of cisplatin resistance in LA cells	(60)
Gastric cancer (GC)	ceRNA network regulation	Sponge effect of miR-515-5p	Promotion of GC cell proliferation, migration, stemness and inhibition of apoptosis	(61)
	ceRNA network regulation	Sponge effect of miR-124-3p and activation of PDK4	Promotion of GC cell growth	(62)
	ceRNA network regulation	Sponge effect of miR-625-5p and activation of NFIX	Promotion of GC cell growth	(63)
	Epigenetic regulation	Activation of SOX4, inhibition of PTEN and activation of PI3K/AKT	Promotion of GC cell growth and migration	(64)
	ceRNA network regulation	Sponge effect of miR-625-5p and activation of STAT3	Promotion of GC cell proliferation and migration	(65)
	ceRNA network regulation	Sponge effect of MiR-124-3p and activation of EZH2	Promotion of GC cell proliferation and invasion	(39)
Colorectal cancer (CRC)	ceRNA network regulation	Sponge effect of miR-153-5p and activation of HIF-1α	Promotion of CRC cell proliferation	(66)
	Epigenetic regulation	HNF4α mediation of the LINC00511/EZH2 axis and inhibition of IL-24	Promotion of CRC cell proliferation and migration	(67)
	ceRNA network regulation	Sponge effect of miR-29c-3p and activation of NFIA	Promotion of CRC cell proliferation, migration and stemness	(68)
	ceRNA network regulation	Sponge effect of miR-625-5p and activation of WEE1	Promotion of CRC cell growth	(69)
Liver cancer (LC) Liver cancer (LC)	ceRNA network regulation	Sponge effect of miR-424	Promote liver cancer cell proliferation and metastasis	(70)
	ceRNA network regulation	Sponge effect of miR-195 and activation of EYA1 expression	Promotion of LC cell proliferation and invasion	(41)
	ceRNA network regulation	Sponge effect of miR-29c	Promotion of LC cell proliferation and migration	(71)
	Induction function	Induction of invasive pseudopod formation and exosome secretion	Promotion of LC invasion	(72)
Osteosarcoma (OS)	ceRNA network regulation	Sponge effect of miR-618 and activation of MAEL	Promotion of OS cell proliferation and migration	(73)
	ceRNA network regulation	Sponge effect of miR-765 and activation of APE1	Promotion of OS cell proliferation and migration	(74)
	–	–	Inhibition of OS cell proliferation and migration	(75)
Pancreatic cancer (PC)	ceRNA network regulation	Sponge effect of miR-29b-3p and activation of VEGFA	Promotion of PC cell proliferation, migration and invasion	(48)
	ceRNA network regulation	Sponge effect of miR-193a-3p and activation of PLAU	Induction of PC cell invasion and migration	(76)
Cervical cancer (CC)	transcriptional regulation	Increased RXRA and upregulation of PLD1	Promotion of CC cell growth	(77)
	transcriptional regulation	Regulation of drug resistance and apoptosis-related genes	Promotion of CC cell apoptosis and reduction resistance to paclitaxel by inhibition of LINC00511	(40)
	ceRNA network regulation	Sponge effect of miR-324-5p and activation of DRAM1	Promotion of CC cell proliferation and invasion	(78)
	ceRNA network regulation	Sponge effect of miR-497-5p and activation of MAPK1	Promotion of CC cell proliferation and invasion	(79)
T-cell acute lymphoblastic leukaemia (TCALL)	ceRNA network regulation	Sponge effect of miR-195-5p and activation of LRRK1	Promotion of TCALL cell migration and proliferation	(80)
Thyroid cancer (TC)	transcriptional regulation	Activation of CDK	Promotion of TC cell proliferation	(81)
	transcriptional regulation	Bound to TAF1 and regulation of JAK2/STAT3	Altered radio-sensitivity of TC cells	(82)
Renal cell carcinoma (RCC)	ceRNA network regulation	Sponge effect of miR-625 and activation of CCND1	Promotion of RCC cell proliferation and migration	(83)
Bladder cancer (BC)	ceRNA network regulation	Sponge effect of miR-143-3p and activation of PCMT1 expression	Promotion of BC cell proliferation and migration	(84)
	transcriptional regulation	Regulation of Wnt/β-cathepsin	Proliferation and apoptosis of BC cell	(85)
Tongue cancer (TC)	ceRNA network regulation	Sponge effect of miR-765 and activation of LAMC2	Promotion of squamous TC cell proliferation and invasion	(86)
Ovarian cancer (OC)	ceRNA network regulation	Sponge effects of miR-424-5p and miR-370-5p	Promotion of OC cell proliferation and migration	(87)
	ceRNA network regulation	Bound to EZH2 and inhibition of P21	Promotion of OC cell proliferation and migration	(47)
Esophageal cancer (EC)	ceRNA network regulation	Sponge effect of miR-150-5p	Promotion of EC cell proliferation, migration and invasion	(88)
Melanoma	ceRNA network regulation	Sponge effect of miR-150-5p and activation of ADAM19	Promotion of melanoma cells migration	(89)

Although it has a relatively complex role in malignant tumors, the regulatory effects LINC00511 can be exerted in three main ways. Firstly, LINC00511 can act as a ceRNA, where it regulates the expression of target genes by interacting with microRNAs (miRNAs) and thereby affecting malignant tumor cells. Secondly, LINC00511 can regulate its own or target gene expression via epigenetic modifications thereby promoting tumor occurrence. Thirdly, LINC00511 can exert effects at the transcriptional level, such as the regulation of target genes and related signaling pathways, thereby influencing tumor progression.

## 4 Expression of LINC00511 in BC

**Figure 3:**
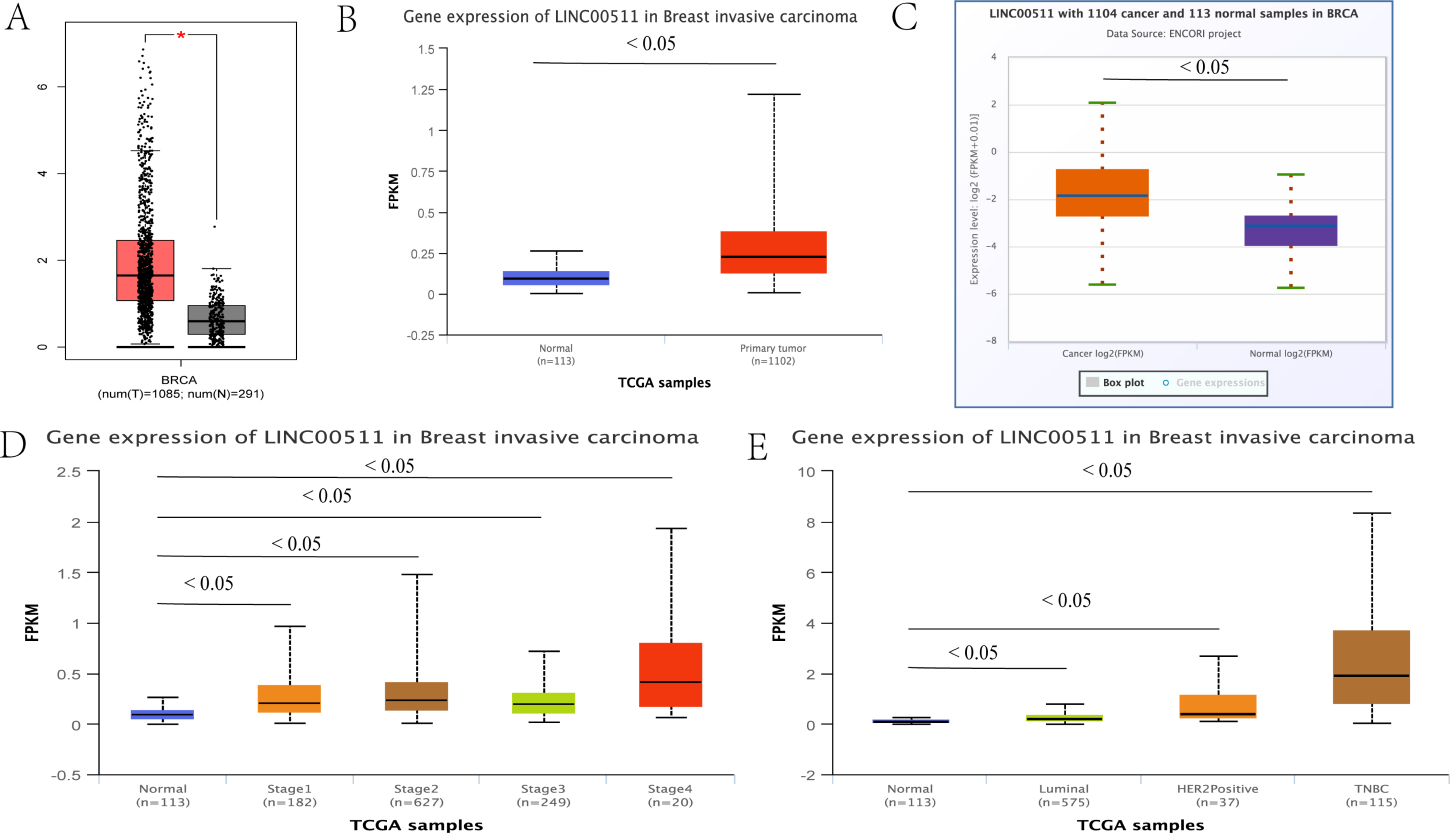
The relationship between the expression levels of LINC00511 in breast cancer and tumor stage and the breast cancer subtype. **(A)** GEPIA, **(B)** UALCAN, **(C)** ENCORI and other online websites were used to show the LINC00511 expression levels in breast cancer. **(D)** The UALCAN online website showed that LINC00511 expression levels were closely related to tumor stage of cancer progression.**(E)** The UALCAN online website showed that LINC00511 expression levels were closely related to the breast cancer subtype.

LINC00511 is known to be dysregulated in various human malignant tumors. In addition, its expression levels is known to be associated with the age patients and their tumor size, stage and subtype as well as their lymph node status. These factors can affect the diagnosis and prognosis of tumor patients, which suggests that it has a role as a potential molecular biomarker (90). By using the publicly available online databases, GEPIA (91), UALCAN (92) and ENCORI (93), LINC00511 was found to be upregulated in BC tissues. The expression levels of this lncRNA were found to be closely correlated to the clinical stage, subtype and subclass of BC (**Figure 3**).

A subsequent study of BC tissue samples confirmed the results obtained from the above online databases. Lu et al. measured LINC00511 expression levels in BC and non-cancerous tissues by qRT-PCR and found that LINC00511 was significantly upregulated in the cancer samples (94). This was confirmed by Tan et al. These authors showed that LINC00511 expression was closely associated with tumor size, clinical stage and lymph node status by using *in vitro* cell cultures (95). Interestingly, another study using serum samples from BC patients also showed that LINC00511 was overexpressed in BC patients where it was found that this lncRNA was significantly associated with the patients’ PR status, ER status, HER2 status, tumor stage, tumor size as well as lymph node status (43).

In summary, several lines of evidence obtained from bioinformatics, blood, tissue and cell experiments, would suggest that there is an overexpression of LINC00511 in BC. Therefore, further research on the regulatory mechanisms of LINC00511 dysregulation in BC would be beneficial for revealing the molecular nature this cancer and this exercise may point to potential new therapeutic targets to combat this disease.

## 5 Related regulatory mechanisms of LINC00511 dysregulation in BC

Currently, the specific regulatory mechanisms underlying the upregulation of LINC00511 expression in BC remain unclear. Studies have shown that the abnormal levels of LINC00511 may affect regulatory mechanisms such as epigenetic modifications. DNA methylation is an epigenetic mechanism that has been proven to play a crucial role in the occurrence and development of BC (96). Liu et al. also confirmed that DNA hypo-methylation in the CpG island region of the gene promoter region was positively and significantly correlated with the levels of LINC00511 expression (97).

The estrogen-signaling pathway is another factor that can affect LINC00511 expression. Zhang et al. explored the relationship between LINC00511 and ER-negative status in a study on ER-negative BC and found that silencing ER expression in cancer cells could induce its expression. Surprisingly, after stimulating BC cells with tamoxifen, an anti-estrogen drug, LINC00511 expression was significantly upregulated. Further studies revealed that estrogen deficiency could directly activate the TF, AP-2 (TFAP-2), thereby upregulating LINC00511 expression in BC cells (98). Currently, only DNA methylation and the estrogen-signaling pathway have been reported to be associated with the regulation of LINC00511 expression in BC. Further research is needed to elucidate the precise mechanisms of action of LINC00511 in BC.

## 6 Molecular regulatory mechanisms of LINC00511

### 6.1 CeRNA networks

LncRNAs often exert their biological functions as ceRNAs. LINC00511 is a typical ceRNA molecule that is capable of base pairing in a complementary fashion with various miRNAs, by competitively binding to them. This prevents the miRNAs from binding to and degrading target genes, thereby regulating target gene expression. Among them, the LINC00511/miR-185-3p/E2F1 axis is a classic ceRNA interaction network. Lu et al. showed that LINC00511 can bind to miR-185-3p through molecular complementarity. This prevents miR-185-3p from binding to its target gene, E2F1, leading to upregulation of E2F1 protein expression and activation of the expression of the downstream TF, Nanog. These interactions promotes the stem-like state and malignant phenotype of BC cells (94). Additionally, LINC00511 can also promote tumor cell proliferation by alleviating the inhibitory effect of miR-150 on matrix metalloproteinase 13 (MMP13) (99). Such typical ceRNA networks can play important roles in the oncogenic functions of LINC00511.

### 6.2 Involvement in transcriptional regulation

Numerous studies have shown that LINC00511 may either directly or indirectly affect the transcription and expression of downstream genes through interactions with TFs, regulatory factors as well as other molecules. Blasiak et al. found that LINC00511 could interact with the vitamin D receptor (VDR), thereby affecting the transcriptional activation process and interfering with the expression of genes related to the vitamin D signaling pathway. This can lead to an anti-BC effect (100). Additionally, Liu et al.’s study also confirmed that LINC00511 could alter the radio-sensitivity of BC cells by regulating the expression levels of STXBP4 (101). This suggests that LINC00511 achieves its biological functions at multiple levels, including gene expression regulation.

### 6.3 Encoding small peptides to regulate signaling pathways

In addition to acting as a classic lncRNA, recent studies have found that LINC00511 may also encode a 133-amino acid small peptide (LINC00511-133aa) resulting in unique biological functions. Tan et al. found that the small peptide encoded by LINC00511 is capable of activating the Wnt/β-catenin signaling pathway. This can promote the invasive capacity and the stem cell state of BC cells (95). This finding provides a novel molecular mechanism for the oncogenic effects of LINC00511 and suggest additional roles for lncRNAs in tumor progression.

### 6.4 Mediating target gene regulation of the immune microenvironment

Recently, it was shown that there is a connection between LINC00511 and tumor immunogenicity and the tumor microenvironment. Sun et al. reported that LINC00511 could regulate the activation of inflammasomes through the LINC00511/miR-573/GSDMC axis, thereby affecting immune cell infiltration and tumor immunogenicity (102). Lian et al. also provided evidence through bioinformatics analysis combined with tissue and cell experiments that LINC00511 participated in regulating immune cell infiltration by modulating the related signaling pathways. They found that LINC00511 could target miR-29-3p, thereby promoting the upregulation of SLC31A1 expression. This molecule then promoted BC progression by regulating tumor immune infiltration (103). These findings suggest that LINC00511 may be involved in remodeling of the immune microenvironment leading to tumor evasion by immune system. This may provide another potential target for tumor immunotherapy.

## 7 Biological functions of LINC00511 in BC

### 7.1 Mediation of tumor cell apoptosis, proliferation, migration and invasion

In several studies, LINC00511 has been shown to significantly promote the proliferation, migration and invasion capabilities of BC cells. Liu et al. found that DNA hypo-methylation could lead to upregulation of LINC00511, thereby promoting BC cell proliferation, invasion and migration (97). LINC00511 could also inhibit cell apoptosis and promote cell proliferation in some *in vivo* experiments where it was found that knocking down LINC00511 could inhibit tumor growth (98). Another study revealed that LINC00511 could positively regulate the expression of MMP13, thereby promoting the migration and invasion of BC cells (99). Therefore, LINC00511 can promote the progression of malignancy in BC by regulating various downstream molecular targets.

### 7.2 Maintenance the tumor stem cell state

Cancer stem cells belong to a small subgroup of tumor cells, which possess self-renewal and tumor-initiating abilities, playing a crucial role in various stages of tumorigenesis, drug resistance, recurrence as well as metastasis. Studies have shown that LINC00511 could positively regulate Nanog expression, thereby maintaining the tumor stem cell-like state and characteristics. Knocking down LINC00511 can significantly downregulate Nanog expression and its downstream genes, inhibiting the formation and self-renewal capacity of tumor stem cells. There is also evidence that LINC00511 may encode a 133-amino acid small peptide (LINC00511-133aa), which can activate the Wnt/β-catenin signaling pathway, and thus maintaining the stem-like state of BC cells (95). Hence, LINC00511 has the ability to regulate the existence and activity of tumor stem cells.

### 7.3 Involvement of LINC00511 in tumor chemotherapy and radiotherapy resistance

Tumor cell resistance to chemotherapeutic drugs and radiation can lead to failure in cancer treatment, recurrence and metastasis. Multiple studies have found that LINC00511 can cause resistance in BC patients. Liu et al. confirmed that LINC00511 could regulate miR-185 expression, thereby affecting the levels of its target gene, STXBP4, ultimately leading to enhanced radio-resistance in BC cells (101). Zhang et al. showed that LINC00511 could enhance cellular resistance to paclitaxel by upregulating the expression levels of CDK6, while knocking down LINC00511 could significantly increase tumor cell sensitivity to this chemotherapeutic drug (104). Wu et al. found that nanomaterial-mediated LINC00511 siRNA delivery technology could significantly improve the chemo-sensitivity of triple negative BC (TNBC) cell lines to cisplatin (105). These findings reveal a key regulatory role of LINC00511 in processes such as chemotherapeutic drug- and radiation-induced tumor cell apoptosis and DNA damage repair.

## 8 Potential clinical applications of LINC00511 in BC

### 8.1 LINC00511 as a molecular biomarker for diagnosis and prognosis of BC

Extensive preclinical and retrospective clinical data have shown that LINC00511 may be used as a potential molecular biomarker for the diagnosis and prognosis of BC. Currently, the main blood biomarkers used in the clinical screening of BC are CEA and CA15-3. However, these have limited diagnostic efficacy for early-stage BC. To find new blood biomarkers in order to improve the diagnostic efficacy of BC, Mahmoud et al. conducted relevant studies on the sera of Egyptian female BC patients (43). They found that, compared to using CEA and CA15-3 alone for diagnosis, a combination screening with either LINC00511/CA15-3 or LINC00511/CEA had higher diagnostic efficacy in distinguishing BC patients from healthy individuals. In addition, LINC00511 also had a certain diagnostic value in the staging and metastasis of BC patients. This study found that the expression pattern of LINC00511 also differed among different molecular subtypes of BC, with a relatively higher expression in TNBC, suggesting its intrinsic association with the occurrence and development of specific subtypes.

LINC00511 has also shown to have a potential value in predicting the survival of BC patients. A systematic review and meta-analysis of LINC00511 in BC patients showed that those with high LINC00511 expression had a shorter overall survival (106). Chen et al. also reached a similar conclusion (107). These findings suggest that LINC00511 may be a potentially valuable molecular marker for BC. Further in-depth studies on LINC00511 will help to optimize the molecular subtyping, prognostic assessment and personalized treatment strategies for BC.

### 8.2 LINC00511 as a therapeutic target for BC

High expression levels of LINC00511 in BC patients precedes a worse survival prognosis with the lncRNA acting as an oncogene in BC. Therefore, knocking out LINC00511 expression in order to inhibit its effects on oncogenes and their related signaling pathways may be an effective new strategy for treatment of BC. Currently, the main targeting strategies for lncRNAs include antisense oligonucleotides, siRNAs (99, 104) and shRNAs (94, 101), which can act as small inhibitor molecules. SiRNA silencing studies by Yuan et al. have involved the construction of a carrier by combining siRNA with cationic nano-bubbles (CNBs). This significantly improved the silencing efficiency of LINC00511 by employing ultrasound-mediated nano-bubble destruction and forming siRNA-CNBs, which were ideal carriers for treating BC (107). Additionally, Wu et al. constructed a novel type of therapeutic diagnostic agent, by using a complex of low-frequency ultrasound irradiation and nano-bubbles, which appeared to be an efficient and safe siRNA transfection strategy (105).

Another method that could be used to inhibit the effects of LINC00511 on oncogenes, involved CRISPR-Cas9-mediated gene editing (108). Azadbakht et al. showed that CRISPR/Cas9 was another potential method for knocking out LINC00511, and subsequent studies have shown that this technique could specifically knock out this lncRNA gene (109). These studies indicate that LINC00511 may a potential novel therapeutic target for BC, and many scholars have proposed and implemented various methods for targeting LINC00511 (38, 110). However, up to now, these practices have only yielded results in cell and animal models, and clinical application studies have not produced satisfactory results. Therefore, further in-depth understanding and elucidation of the functional mechanisms of LINC00511 are needed, followed by the development of targeted drugs, to bring new treatment options for BC patients.

## 9 Prospects and challenges

**Figure 4:**
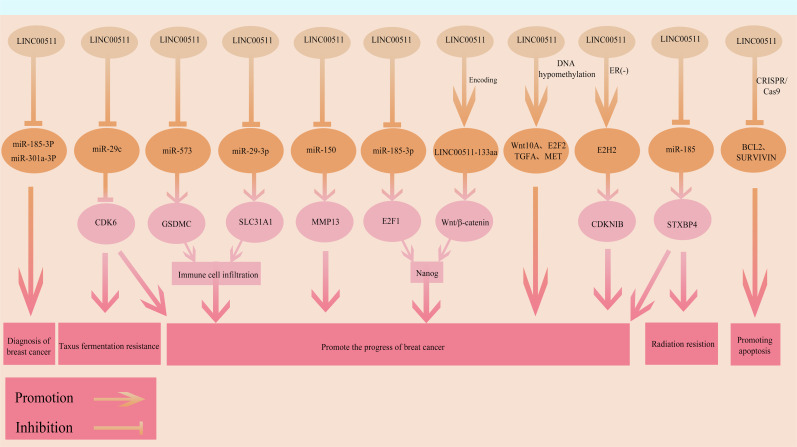
The functional roles and regulatory mechanisms of LINC00511 in BRCA. The arrow and blocking symbols represent promotion and inhibition of molecules/events, respectively.

LINC00511 is a functionally diverse and broad acting oncogenic lncRNA in BC, participating in multiple key biological processes in these cells (**Figure 4**). Its mechanisms of action involve ceRNA networks, transcriptional regulation, signaling pathway activation as well as small peptide encoding at multiple levels. New functions and regulatory mechanisms of LINC00511 in BC are being discovered. However, there are currently several gaps in our understanding of the overall mechanism of action of LINC00511 in tumor occurrence and development, as well as its associated networks with other molecular events. Additionally, the precise mechanisms by which LINC00511 regulates downstream targets and signaling pathways, and its differential roles at different time points in BC remain to be further explored. With respect to its clinical applications, LINC00511 may be used to improve prognostic assessment for BC, although further studies are urgently needed.

In conclusion, in-depth elucidation of the functions and mechanisms of action LINC00511 will not only expand our understanding of the molecular networks underlying tumor occurrence and development, but will also provide new insights for early diagnosis, molecular subtyping and clinical treatment of BC. The study of lncRNAs and in particular, LINC00511 and its regulatory networks, has the potential to deliver novel breakthroughs in the precise and personalize diagnosis and treatment of all cancers.
